# The role of macrophages polarization in predicting prognosis of radically resected gastric cancer patients

**DOI:** 10.1111/jcmm.12109

**Published:** 2013-11-27

**Authors:** Francesco Pantano, Pierpaolo Berti, Francesco Maria Guida, Giuseppe Perrone, Bruno Vincenzi, Michelina Maria Carla Amato, Daniela Righi, Emanuela Dell'Aquila, Francesco Graziano, Vincenzo Catalano, Marco Caricato, Sergio Rizzo, Andrea Onetti Muda, Antonio Russo, Giuseppe Tonini, Daniele Santini

**Affiliations:** a Department of Medical Oncology, Campus Bio-Medico University of RomeRome, Italy; b Department of Hematology, Campus Bio-Medico University of RomeRome, Italy; c Department of Anatomical Pathology, Campus Bio-Medico University of RomeRome, Italy; d Department of Onco-Hematology, Azienda Ospedaliera San SalvatorePesaro, Italy; e Department of Surgery, Campus Bio-Medico University of RomeRome, Italy; f Department of Surgical, Oncological and Stomatological Sciences, University of PalermoPalermo, Italy

**Keywords:** tumour associated macrophages, M1 polarization, M2 polarization, prognostic factor, gastric cancer

## Abstract

Tumour-associated Macrophages (TAM) present two different polarizations: classical (M1) characterized by immunostimulation activity and tumour suppression; alternative (M2) characterized by tumour promotion and immune suppression. In this retrospective study, we evaluated the correlation between the two forms of TAM with survival time in radically resected gastric cancer patients. A total of 52 chemo- and radio-naive patients were included. Two slides were prepared for each patient and double-stained for CD68/NOS2 (M1) or CD68/CD163 (M2) and five representative high-power fields per slide were evaluated for TAM count. The median value of the two macrophage populations density and the median value of M1/M2 ratio were used as cut-off. Twenty-seven patients with M1 density above-the-median had a significantly higher survival compared to those below the median. Twenty-six patients with M1/M2 ratio above the median showed median OS of 27.2 months compared to 15.5 months of the patients below the median. No association between M2 macrophage density and patient's outcome was found. In multivariate analysis, M1/M2 was a positive independent predictor of survival. The M1 macrophage density and M1/M2 ratio, as confirmed in multivariate analysis, are factors that can help in predicting patients survival time after radical surgery for gastric cancer.

## Introduction

Tumour-associated macrophages (TAMs) represent a substantial fraction of the growing tumour mass and are associated with poor prognosis in several human cancers 1. TAMs exist in two different polarizations classified as M1 and M2. M1 macrophages show a protective role in tumorigenesis activating tumour-killing mechanisms and antagonizing the activities of different M2 macrophages that are clearly involved in suppression of adaptive tumour-specific immune responses and in promotion of tumour growth, invasion, metastasis, stroma remodelling and angiogenesis 2–6. Unfortunately, the majority of TAM exhibit characteristics of M2 polarization 7–8. M1 macrophages differentiation is induced by interferon-γ, lipopolysaccharides, tumour necrosis factor (TNFα) and granulocyte–monocyte colony-stimulating factor and are phenotypically characterized by high levels of interleukin IL-12, IL-23, TNFα, IL-1, IL-6, granulocyte-–macrophage colony-stimulating factor (GM-CSF), CXC ligand 10 (CXCL10), inducible nitric oxide synthase (iNOS), human leucocyte antigen (HLA)-DR, reactive oxygen and nitrogen intermediates. M2 macrophages differentiation is induced by IL-4, IL-10, IL-13, IL-21, activin A, immune complexes, and glucocorticoid and express high levels of IL-10, IL-1 receptor antagonist, CC ligand 22 (CCL22), scavenger, mannose and galactose receptors, arginase I and CD163 antigen 7–14.

Specifically, it has been reported that TAM infiltration into tumour tissue correlates significantly with tumour vascularity in human oesophageal and gastric cancers 15 and it is also has been found a direct association between the degree of TAM infiltration and depth of tumour invasion, nodal status and clinical stage in gastric cancer 16. Nevertheless, no study evaluated the correlation between M1/M2 tumour infiltration and the overall survival (OS) in gastric cancer patients. Against these backgrounds, we have decided to evaluate the prognostic role of TAM infiltration in patients affected by radically resected gastric cancer.

## Materials and methods

### Study population

This study was approved by the Institutional Review Board of Campus Bio-Medico University, Rome, Italy. The procedures to obtain human gastric cancer tissues and follow-up information are in accordance with the Ethical Principles for Medical Research Involving Human Subjects as formulated in the World Medical Association Declaration of Helsinki (revised in 2008). All specimens were retrospectively obtained from the archives of formalin-fixed, paraffin-embedded tissue blocks in the Departments of pathology of Campus Bio-Medico University, Rome, and of Medical University of Pesaro-Urbino, Italy. The gastric cancer tissues were collected from surgeries performed from May 2000 to August 2004. The patients were followed up until December 2011 through outpatient visits and/or correspondences to family members. The inclusion criteria were complete follow-up data, paraffin blocks available, R0 radical surgery, pre-operative chemotherapy or radiotherapy excluded. All of the cases that satisfied the inclusion criteria were included in this study. The patients were selected without knowledge of their previous tumour macrophage counts. Histological evaluation was based on the World Health Organization criteria 17. Pathological findings (tumour size, spread and lymph-node status) were obtained from the pathologist's original reports. Tumour–node–metastasis status (TNM) classification was reassessed using seventh edition of the UICC/AJCC classification 18.

### Statistical analysis

The OS time was calculated as the period from the date of surgery until death. OS was determined by Kaplan–Meier product-limit method. Moreover, the differences in terms of OS according to the prognostic variables were evaluated by the log-rank test. Finally, the Cox proportional hazards model was applied to the multivariate survival analysis 19. SPSS software (version 19.00, SPSS, Chicago, IL, USA) was used for statistical analysis. A *P* value of less than 0.05 was considered to indicate statistical significance.

### Immunofluorescence

The gastric cancer tissue specimens of 52 patients who had undergone resection for curative intent were analysed. All sections contained tumour and peritumoural tissue. Consecutive 3 μm sections were cut from each block for immunofluorescence experiments. To evaluate M1 and M2 macrophages population, mouse anti-human macrophage CD68 mAb (dilution 1:50; clone PG-M1, DAKO, Hamburg, Germany), CD163 rabbit monoclonal mAb (dilution1:200; clone 10D6, Novocastra, Newcastle upon Tyne, UK) and NOS2 rabbit polyclonal antibody (N-20) (dilution 1:400; Santa Cruz Biotecnology Inc., Santa Cruz, CA, USA) were adopted. Incubation with primary antibodies against CD68 was followed by Alexa Fluor 488 (dilution 1:200; goat antimouse; Invitrogen, USA); NOS2 and CD163 was followed by and Alexa Fluor 568 (dilution 1:200; donkey anti-rabbit Invitrogen, Darmstadt, Germany). A mounting medium containing DAPI (Vectashield, Vector Laboratories, Burlingame, CA, USA) was used. Negative control slides processed without primary antibodies were included for each staining. Thus, two slides were prepared for each patient and double-staining of CD68/NOS2 (markers for M1 macrophages) and CD68/CD163 (markers for M2 macrophages) was performed.

### Analysis and validation of immunostaining

**Figure 1:**
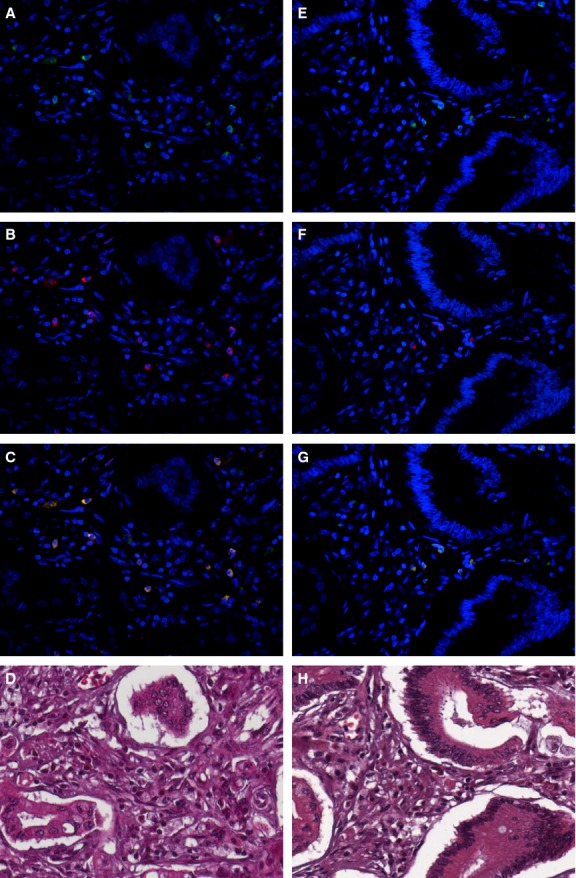
Immunohistochemistry of macrophage in gastric cancer. Gastric cancer stained for M1 (A–C) and M2 (E–G) immunofluorescence pattern. (A, E) Nucleated cells positive staining for CD68 (green), which resulted in cytoplasm staining of macrophages. (B) INOS staining (red) in some nucleated cells. (C) Merged image of DAPI (blue), CD68 (green) and INOS (red) defining M1 macrophages (green/red double staining). (F) CD163 staining (red) in nucleated cells. (G) Merged image of DAPI (blue), CD68 (green) and CD163 (red) defining M2 macrophages (green/red double staining). (D, H) E&E images of immunofluorescence fields. Original magnification 400×.

Five representative high-power fields (×400 magnification) of the deepest infiltrative tumour areas per tissue section were selected using an Eclipse 80i Nikon microscope (Nikon, Tokyo, Japan). Acquisition was carried out using the Imaging Software NIS-Elements (Nikon). Cell counts were performed on collected composite images, in which the signal from the fluorochrome had been assigned a different pseudo-colour (green for 488, red for 568 and blue for DAPI). Adobe Photoshop CS 5 (version 5.5) software was used to generate composite images from three different fluorochrome signals. The number of nucleated cells with positive staining for the phenotype marker in each image was then counted manually. Evaluation was performed simultaneously by two investigators (MA, PB) who were blinded as regards which group the specimens belonged to and to patient outcome. The investigators analysed M1 macrophages, M2 macrophages and the M1/M2 ratio counting the absolute macrophage number for all high-power field per section (Fig. 1).

## Results

### Patient characteristics

Fifty-two patients were included in this retrospective study. All of the patients had complete follow-up information and the pathological diagnosis was confirmed by a pathologist prior to inclusion in this study. No patients received chemotherapy and/or radiotherapy before or after surgery. The overall cumulative survival rates were 77% (40 patients) for 1 year, 42% (22) for 2 years, 15% (8) for 3 years, 13% (7) for 4 years and 11% (6) for 5 years. The clinicopathological characteristics were summarized in Table 1.

**Table 1:** Clinicopathological characteristics (*n* = 52)

**Median survival (months; Mean ± SD)**	**21.5 (27.45 ± 25.56)**
**Age (years; Mean ± SD)**	**61.7 ± 1.2**
**Gender (male:female)**	**23:29**
**Tumour stage, number (%)**	
** Ia**	**1 (2)**
** Ib**	**3 (5)**
** IIa**	**18 (34)**
** IIb**	**9 (17)**
** IIIa**	**9 (17)**
** IIIb**	**12 (23)**
**Histology, number (%)**	
** Intestinal**	**29 (55)**
** Diffuse**	**10 (19)**
** Mix**	**9 (17)**
** Poorly differentiated**	**4 (7)**
**Tumour grade, number (%)**	
** G1**	**5 (9)**
** G2**	**17 (33)**
** G3**	**28 (54)**
** Not recorded**	**2 (4)**

### Macrophage distribution

In the five representative high-power fields (×400 magnification) per tissue section of the 52 patients, the median cell count value of CD68/NOS2 macrophages (markers for M1 polarization) was 7.0 (mean 7.4 ± 3.4; CI: 6.5–8.3) whereas the median cell count value of CD68/CD163 (markers for M2 polarization) was 6.4 (mean 7 ± 3.9; CI: 5.9–8.1). In addition, M1/M2 ratio was calculated. The median value of M1/M2 ratio between the median cell count value of the two macrophage populations was 1.16 (mean 1.26 ± 0.70; CI: 1.07–1.45).

### Correlation between the M1/M2 macrophage density and the M1/M2 ratio with clinicopathological characteristics

We found that there was no statistically significant association between the M1 macrophage density, M2 macrophage density and M1/M2 ratio with clinicopathological characteristics as tumour stage, histology, lymphonode invasion and grade (*P* > 0.05).

### Correlation between the M1/M2 macrophage density and the M1/M2 ratio with survival time

**Figure 2:**
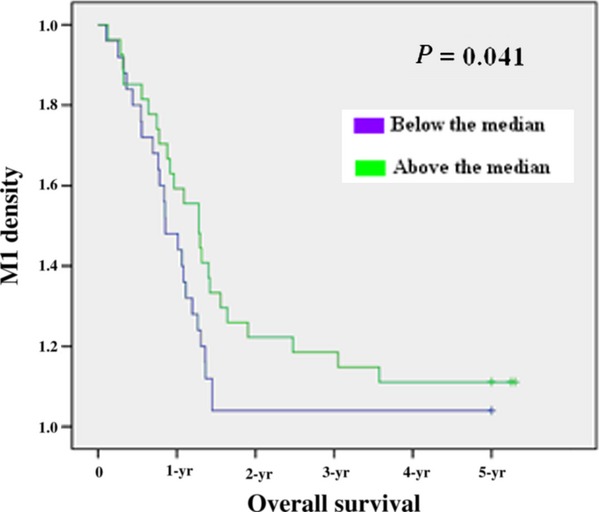
Kaplan–Meier 5-year survival curves according to M1 polarization.

**Figure 3:**
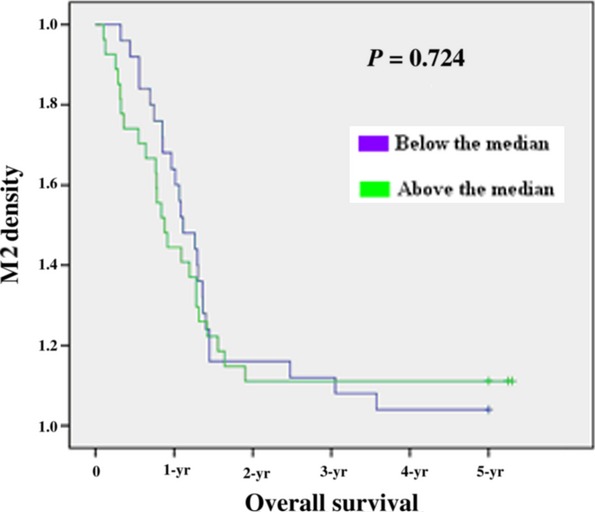
Kaplan–Meier 5-year survival curves according to M2 polarization.

**Figure 4:**
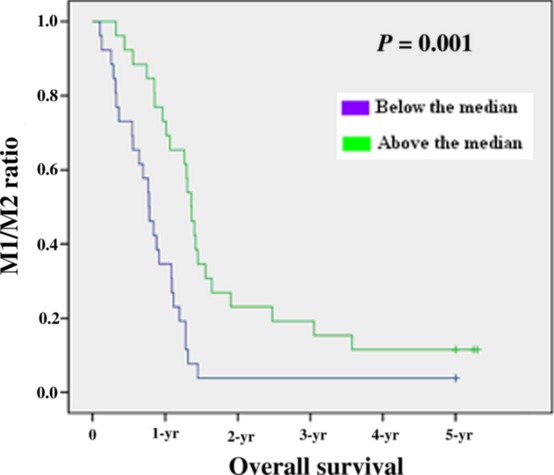
Kaplan–Meier 5-year survival curves according to M1/M2 ratio.

To assess whether there is any value of the macrophage density of M1 and M2 in predicting prognosis, the median value of the macrophage density of two populations was used as a cut-off point to dichotomize the 52 patients into a group with a macrophage density above or below the median value according to previous studies 20–21. Kaplan–Meier survival curves were plotted to investigate further the association of cell densities with survival. The log-rank test was used to compare survival rates. We found that the 27 patients with above-the-median M1 macrophage density had a 1-year survival rate of 81% (22 patients), 2-year survival rate of 55% (15), 3-year survival rate of 26% (7), 4-year survival rate of 22% (6) and 5-year survival rate of 18% (5) with median survival of 25.6 months, which were significantly higher than the corresponding survival rates 72% (18), 24% (6), 4% (1), 4% (1) and 4% (1) with Median Survival of 17.1 months, respectively, in the 25 patients with below-the-median M1 macrophage density, with *P* = 0.041 (*P* < 0.05) (Fig. 2, Table 2). In contrast, the 27 patients with above-the-median M2 macrophage density had a 1-year survival rate of 70% (19), 2-year survival rate of 37% (10), 3-year survival rate of 15% (4), 4-year survival rate of 11% (3) and 5-year survival rate of 11% (3) with median survival of 17.6 months, which were lower but not significantly than the corresponding survival rates 84% (21), 48% (12), 16% (4), 16% (4) and 12% (3) with median survival of 22.3 months, respectively, in the 25 patients with below-the-median M2 macrophage density, with *P* = 0.724 (*P* > 0.05) (Fig. 3). The median value of the M1/M2 ratio was used as a cut-off point to dichotomize the 52 patients into a group with a M1/M2 ratio above or below the median. The 26 patients with above-the-median M1/M2 ratio had a 1-year survival rate of 88% (23), 2-year survival rate of 65% (17), 3-year survival rate of 27%(7), 4-year survival rate of 23% (6) and 5-year survival rate of 15% (4) with median survival of 27.2 months, which were significantly higher compared with the corresponding survival rates of 65% (17), 19% (5), 3% (1), 3% (1) and 3% (1), respectively, in the 26 patients with below-the-median M1/M2 ratio with Median Survival of 15.5 months with *P* = 0.001 (*P* < 0.05)(Fig. 4, Table 2). To determine whether the macrophage density is independently associated with patient's survival time, the multivariate Cox proportional hazards analysis was used. Extent of the tumour (T), spread to regional lymph nodes (N) and differentiation grade (G) were included in the multivariate analysis along with macrophage density M1 and the M1/M2 ratio. In the multivariate analysis, we found that only the M1/M2 ratio was a positive independent predictor of patient's survival time (*P* = 0.001; hazard ratio 0.410) (Table 2). The M1 macrophage densities had no statistically significant association with patient's survival time in the multivariate analysis (*P* > 0.05).

**Table 2:** Correlation between the M1 density and M1/M2 ratio and overall survival

Macrophage density	Patients	Survival rate (%)					Median survival months (CI; ±SD)	*P*	
	*N*	1-year	2-years	3-years	4-years	5-years		Univariate	Multivariate
M1									
Above the median	27	81	55	26	22	18	25.6 (22.33–44.85; ±29.86)	0.041	0.314
Below the median	25	72	24	4	4	4	17.1 (13.66–27.98; ±18.28)		
M1/M2 ratio									
Above the median	26	88	65	27	23	15	27.2 (25.45–47.51; ±28.72)	0.001	0.001
Below the median	26	65	19	3	3	3	15.5 (11.36–25.50; ±18.40)		

CI: confidence interval; n: number; SD: standard deviation.

## Discussion

Macrophages are one of the major populations of tumour-infiltrating immune cells. In most of the solid tumours, however, the existence of TAM is advantageous for tumour growth and metastasis 1. It is demonstrated that tumour escape has been linked with a switch from M1 activation in the early tumour initiation process towards M2-like phenotype during tumour progression 22. M1 and M2 subsets differ in terms of phenotype and functions. M1 cells have high microbicidal activity, immuno-stimulatory functions and tumour cytotoxicity. On the other hand, M2 cells produce interleukin 10 (IL-10) and transforming growth factor β (TGF- β) leading to a suppression of general antitumour immune responses, promoting tumour neoangiogenesis by the secretion of pro-angiogenic factors and defining the invasive microenvironment to facilitate tumour metastasis and dissemination 23. Anyway, at present, there is conflicting evidence regarding the role of tumour macrophage infiltrate in influencing patients survival. In many human neoplasms including lung, breast, cervix, bladder, ovary and pancreas cancers, the presence of extensive TAM infiltrate correlates with poor prognosis. In other tumours, including those of the brain and prostate, there is conflicting evidence regarding the role of macrophages in survival outcome 1–27. The basis for these conflicting data may be explained considering that in these studies tumour-associated macrophages were detected only by the immunohistochemical analysis of CD68^+^ cells. Matter of fact, CD68 expression is shared between M1 and M2 phenotypes and the use of CD68 as sole could not represent a reliable marker in evaluating the real impact of the two subtypes with almost opposite biological properties. Only two studies have analysed the relationship between M1 and M2 macrophages and prognosis using a double IHC staining to better characterize the two subsets of TAM. These reports demonstrated a significant direct correlation between M1 phenotype infiltrate and the survival time of patients affected by non-small cells lung cancer (NSCLC) 20–21. In our study, we used double-staining for CD68/NOS2 as markers for M1 macrophages and CD68/CD163 as markers for M2 macrophages to be in accordance with the most part of previously published studies that performed a phenotypic characterization of macrophages polarization 20–34. The haemoglobin scavenger receptor, CD163, is expressed on tissue macrophages and monocytes after M2 polarization 35. Conversely, macrophages M1 polarized by exposure to interferon (IFN)-γ or LPS up-regulate inducible nitric oxide synthase (iNOS) to convert into nitric oxide that combining with oxygen radicals lead to the formation of cytotoxic peroxynitrite 36. These markers are not absolutely specific, for example CD68 has been found in immature CD1a-positive dendritic cells 37–38, CD163 is also expressed in some dendritic cells 39, and iNOS is expressed by endothelial cells 40 as well as by arterial wall smooth muscle cells 41. For these reasons, we paid particular attention to cell morphology to minimize these potential bias: in this direction we used DAPI as nuclear morphological marker and we counted as positive merely nucleate cell avoiding possibility to count the same cell more than once.

At present, gastric cancer has poor overall survival at 5 years. The therapeutic approach is multidisciplinary and the surgery plays a central role 42, even if the overall survival after radical surgery remains poor. Only 29% of patients undergoing a D2 linfectomy and 21% of those undergoing a D1 linfectomy are alive at 15 years from surgery. Moreover, radical surgery in gastric cancer is often associated with important comorbidities 43. The discovery of new and reliable bio-markers able to select patients who really benefit from surgery is an urgent need in clinical practice.

Currently, the only proven prognostic indicators are represented by clinical-pathological factors such as age, sex, gastric wall infiltration, locoregional nodal involvement, Laurens' histology and margins. Furthermore, in the last years, a growing attention was paid to new molecular prognostic markers related to cancer cell biology while few data are available about the role of tumour microenvironment in predicting patient's outcome. Two recent works demonstrated that gastric cancer patients with an high TAM count showed poorer surgical outcome than those with a low TAM count 16–44. On the contrary, this is the first study investigating the two forms of macrophages population in gastric cancer. We demonstrated that the M1 macrophage density is a prognostic factor in univariate analysis in accordance with previous reports on the association of M1-survival time in patients affected by non-small cells lung cancer 20–21. More interestingly, in our experience, only the M1/M2 ratio was an independent prognostic factor. Therefore, the cellular and molecular interactions between M1 and M2 population appear to play a major role in determining prognosis of gastric cancer patients. These data support future larger prospective confirming studies investigating the prognostic role of TAM polarization in radically resected gastric cancer with the aim to discriminate patients who could take the most advantage from surgery. Finally, these results highlight new therapeutic horizons involving strategies aiming to reverse TAM phenotype in gastric cancer.
